# Evaluation of dialysis centres: values and criteria of the stakeholders

**DOI:** 10.1186/s12913-020-05085-w

**Published:** 2020-04-14

**Authors:** Eduardo Parra, María Dolores Arenas, María José Fernandez-Reyes Luis, Angel Blasco Forcén, Fernando Alvarez-Ude, Juan Aguarón Joven, Alfredo Altuzarra Casas, José María Moreno-Jiménez

**Affiliations:** 1 Hospital Universitario Miguel Servet, Zaragoza, Spain; 2 Hospital del Mar, Barcelona, Spain; 3 Complejo Asisntencial Segovia, Segovia, Spain; 4 Universidad de Zaragoza, Zaragoza, Spain

**Keywords:** Delivery of health care, Health care quality assessment, Renal dialysis, Social values

## Abstract

**Background:**

Evaluation of renal replacement therapy with haemodialysis is essential for its improvement. Remarkably, outcomes vary across centres. In addition, the methods used have important epistemological limitations, such as ignoring significant features (e.g., quality of life) or no relevance given to the patient’s perspective in the indicator’s selection. The present study aimed to determine the opinions and preferences of stakeholders (patients, clinicians, and managers) and establish their relative importance, considering the complexity of their interactions, to facilitate a comprehensive evaluation of haemodialysis centres.

**Methods:**

Successive working groups (WGs) were established using a multicriteria methodology. WG1 created a draft of *criteria and sub-criteria*, WG2 agreed, using a qualitative structured analysis with pre-established criteria, and WG3 was composed of three face-to-face subgroups (WG3-A, WG3-B, and WG3-C) that weighted them using two methodologies: weighted sum (WS) and analytic hierarchy process (AHP). Subsequently, they determined a preference for the WS or AHP results. Finally, via the Internet, WG4 weighted the criteria and sub-criteria by the method preferred by WG3, and WG5 analysed the results.

**Results:**

WG1 and WG2 identified and agreed on the following evaluation criteria: evidence-based variables (EBVs), annual morbidity, annual mortality, patient-reported outcome measures (PROMs), and patient-reported experience measures (PREMs). The EBVs consisted of five sub-criteria: type of vascular access, dialysis dose, haemoglobin concentration, ratio of catheter bacteraemia, and bone mineral disease. The patients rated the PROMs with greater weight than the other stakeholders in both face-to-face WG3 (WS and AHP) and WG4 via the Internet. The type of vascular access was the most valued sub-criterion. A performance matrix of each criterion and sub-criterion is presented as a reference for assessing the results based on the preferences of the stakeholders.

**Conclusions:**

The use of a multicriteria methodology allows the relative importance of the indicators to be determined, reflecting the values of the different stakeholders. In a performance matrix, the inclusion of values and intangible aspects in the evaluation could help in making clinical and organizational decisions.

## Background

Since the development of dialysis in the 1960s, the treatment of end-stage renal disease with dialysis has been a challenge worldwide. Less than 0.015% of the population is estimated to be on dialysis, but consumes approximately 2% of healthcare expenditure [1]. In Europe alone, more than 180,000 people are undergoing renal replacement therapy with haemodialysis in more than 4000 centres. The estimated cost of such treatment is between 30,000 and 47,000 euros per patient per year [2, 3].

Importantly, the results of treatment with haemodialysis vary between centres in both the USA and Europe. The *Dialysis Outcomes and Practice Patterns Study* (DOPPS) detected between the different facilities of the USA a variability in the adjusted mortality of almost double (88.7%), in the transfusions performed of more than double (113.9%), and in the prevalence of autologous fistulas of more than 50% (56.0%) [4]. Significant differences were also detected among seven European countries with regards to compliance with clinical guidelines [5].

On the other hand, assessment of dialysis outcomes is generally based on partial methodologies that exclude relevant features (e.g., quality of life, satisfaction, or costs) or present biases (e.g., they do not consider the perspective of the stakeholders, such as patients and managers) [6, 7]. A substantial epistemological limitation of evidence-based medicine has been suggested to be that its indicators reflect the preferences of researchers, ignoring those of other stakeholders [8]. The methods for evaluating health outcomes should consider aspects associated with the individuals involved, such as prioritizing patient-centred care, procuring their welfare, incorporating stakeholder participation in the evaluation, security, transparency, and dignity, respect, and compassion [9–12].

In addition, health organizations are characterized by a large number of dynamic components that, in the real world, interact in complex ways through frequently unpredictable relationships. The evaluation of clinical results from a traditional perspective that ignores these complex relationships is insufficient [13]. In this sense, it is necessary to develop new evaluation methodology that is more realistic and effective, and that considers the complexity of health organizations.

Multicriteria decision analysis (MCDA), also known as the multicriteria decision, includes a set of approaches capable of improving decision-making in complex systems and has been recommended by the International Society for Pharmacoeconomics and Outcomes Research, Health Science Policy Council. Multicriteria methods allow the values and preferences of the stakeholders to be captured, integrating their different perspectives, adding the information in a single expression value, and doing it transparently, consistently, and legitimately [14–18].

The present study aimed to determine the opinions and preferences of the stakeholders in the treatment with haemodialysis, to determine indicators of their results, and to establish their relative importance following a multicriteria approach. This knowledge would allow the creation of an instrument based on values, effectively enabling assessment of the results of different centres, their comparison, and then using it to improve them.

## Methods

For the multicriteria study of stakeholder preferences, five working groups (WGs) were created consecutively, each with specific objectives. All of them were face-to-face, except for WG4, which was via the Internet.

**Fig. 1:**
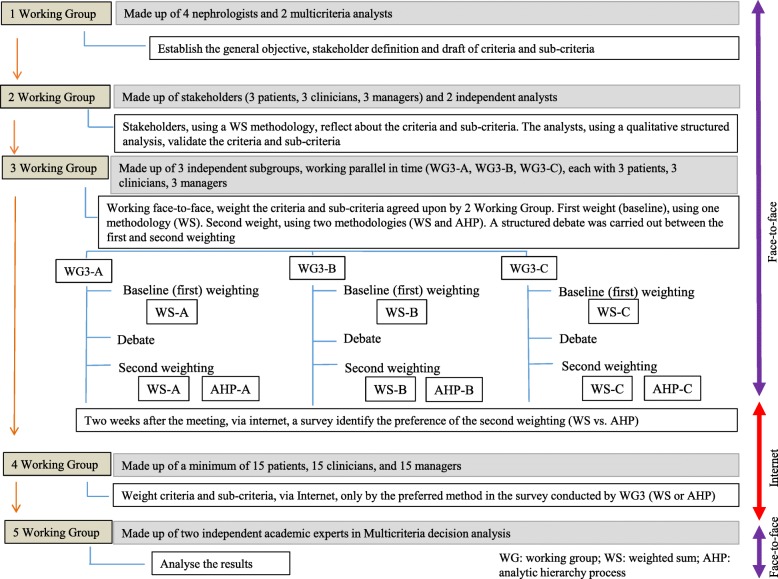
The figure shows the working groups, their composition, flows, methodologies and objectives

WG1 defined the general objectives, identified the groups of “stakeholders” or relevant actors that provide the preferences, and created a draft of criteria and sub-criteria. WG2 evaluated and agreed on the criteria and sub-criteria. WG3 was comprised of three subgroups: WG3-A, WG3-B, and WG3-C. Each of these groups independently, in parallel, and face-to-face weighted the criteria and sub-criteria according to their preferences using two different multicriteria methodologies: weighted sum (WS) and analytic hierarchy process (AHP). Two weeks after this weighting, a survey was sent by email to each individual regarding their preference for the results of the WS or AHP method. Via the Internet, WG4 weighted the criteria and sub-criteria only by the method with the highest preference in the survey. In this way, a new weighting of the criteria and sub-criteria was established through similar methodology, but with a larger sample in order to validate the results of the face-to-face WG3 (obtained with a small sample size) and guaranteeing significant conclusions. Finally, WG5 consisted of two independent academics, specialists in multicriteria, who analysed the results (Fig. 1).

### Working group 1

WG1 consisted of six researchers: four were nephrologists and two multicriteria analysts. The group defined the general objective of the study and the structure and composition of the remaining groups. The general objective was to determine the relevant criteria and sub-criteria of haemodialysis treatment and their weighting according to the preferences of the stakeholders. The preferences of the stakeholders allow a “performance matrix” summarizing the measured preferences for each relevant criteria to be established and determine an “aggregation function” that allows weights to be combined consistently with stakeholders’ preferences. This function enables analysis of the results of the centres considered in the study, and establishes their individual qualification in an orderly and justified manner.

WG1 defined the requirements and number of actors that comprised WG2, WG3, and WG4, which included patients, clinicians, and managers. All participants were recruited on a voluntary basis. The patients should have been in haemodialysis at least three years and have exercised coordination tasks in some organization of kidney patients. They were recruited from such organizations, mainly ALCER (*Asociación para la Lucha Contra las Enfermedades Renales*). The clinicians had to be of recognized prestige and extensive experience, one of them a nephrologist, an internist, and a nurse. For the managers, three profiles were defined that should be present in each group: economic direction, medical direction, and health services researcher. Clinicians and managers were recruited mainly from the centres involved in the study, they were contacted via phone-call, e-mail or personal approach. WG2 and WG3 (WG3-A, WG3-B, and WG3-C) were each comprised of nine interested individuals: three patients, three clinicians, and three managers (total 36 individuals: 4 groups × 9 interested in each). WG3-A was located in Alicante, WG3-B in Segovia, and WG3-C in Zaragoza. WG4 was comprised of at least 15 stakeholders from each category (patients, clinicians, and managers) who were located in different parts of Spain and participated online.

The criteria and sub-criteria were identified sequentially in two steps. First, WG1 agreed on the draft criteria, and then WG2 agreed on the criteria. The criteria are the relevant factors for the evaluation and ordering of the different options (haemodialysis centres). These must meet certain requirements in relation to the MCDA methodology used (completeness, non-redundancy, non-overlap, and preference independence). The following principles were also considered for the selection of draft criteria: feasibility of its implementation, potential modifiability of the indicator, and impact on the patient.

WG1 defined the search strategy in PubMed/Medline, EMBASE, and Cochrane Library. The terms included were: *haemodialysis, outcomes, registry, patient reported outcomes* (and equivalents), and *clinical guideline*. Priority was given to the PRISMA clinical guidelines (Preferred Reporting Items for Systematic Reviews and Meta-analyses). Two WG1 members independently reviewed the literature results and proposed a first draft of the criteria and sub-criteria to the rest of the group. After a discussion in the whole group, the draft criteria and sub-criteria were approved. An “evidence-based” criterion composed of various sub-criteria was established. To determine this, the group decided to consider only the recommendations of level 1 in international clinical guidelines to provide the study with transparency and reproducibility. This decision was made in a manner consistent with the GRADE (Grading of Recommendations Assessment, Development, and Evaluation) considerations. We focus on three clinical guidelines that provide an appropriate framework for the study: *Kidney Disease Improving Global Outcomes* (KDIGO, https://kdigo.org), *European Renal Best Practice* (ERBP, http://www.european-renal-best-practice.org) and *Kidney Disease Outcomes Quality Initiative* (KDOQI, https://www.kidney.org). Finally, the ultimate decision on the inclusion of each criterion was made separately by the majority of the group (at least four members of WG1).

### Working group 2

WG2 consisted of stakeholders (3 patients, 3 clinicians, and 3 managers) working face-to-face. The group used WS methodology to reflect the criteria and sub-criteria by carrying out a qualitative structured analysis of the draft criteria and sub-criteria prepared by WG1. The deliberation was recorded and two independent analysts from the group with four pre-established criteria (internal, external validity, reliability, and objectivity) contributed to validating the selected criteria.

### Working group 3

The face-to-face WG3 weighted the criteria and sub-criteria agreed upon by WG2. WG3 consisted of three subgroups, independent and parallel in time (WG3-A, WG3-B, WG3-C). Following a multicriteria approach, the following were performed sequentially: baseline weighting using the WS methodology, a structured debate, and a second weighting using two different multicriteria methodologies: WS and AHP. The purpose of the weighting was to elicit the preferences of the stakeholders for each of the criteria and the reasons for their preferences.

The WS is an additive model in which the stakeholder is invited to distribute 100 points proportionally to his preferences among the set of criteria and sub-criteria (total sum 100). For example, if stakeholder *Patient 1* has to rank a group of four criteria according to his preferences, distributing 100 points among them, the weight could be 10 points for criteria 1, 60 for criteria 2, 10 for criteria 3, and 20 for criteria 4. The stakeholder establishes the weights for each criterion simultaneously. The AHP is a multicriteria technique in which, for each node of the hierarchy considered, the stakeholder compares in pairs the relative importance of the elements (criteria, sub-criteria, or alternatives) that hang from it according to the fundamental scale of Saaty [19]. The result in each node is a positive reciprocal square matrix from which the local priorities are obtained and a measure of the decision-maker’s inconsistency when issuing their judgments. The *Super Decisions* program was used for this (https://www.superdecisions.com, sponsored by Creative Decisions Foundation). The results obtained are transferred in the same way to a distribution of 100 points to their preferences among all of the criteria and sub-criteria (total sum 100 points). For example, in the AHP model, if stakeholder *Patient 1* weighs a group of four criteria using a pairwise consecutive comparison (criterion 1 with 2, criterion 1 with 3, criterion 1 with 4, criterion 2 with 3, criterion 2 with 4, and criterion 3 with 4), the comparison is made on a quantitative scale that reflects the importance of one criterion in relation to the other. Both criteria can be ranked equally. The *Super Decisions* program allows the weight of each criterion to be found, in which the sum of all criteria is 100 (e.g., 10 points to criterion 1, 60 to criterion 2, 10 to criterion 3, and 20 to criterion 4). In this way, the results obtained by the WS and AHP methods are comparable. The assessment of criteria requires a personal and collective reflective process based on the individual weighting and a collective structured debate of the stakeholders, reasoning their different interests and perspectives to outline the trade-offs between criteria.

Two weeks after the meeting of each WG3, a survey was carried out with each participant. They were asked which results (WS vs. AHP) better reflected their preferences (or none of them or both equally). The survey was conducted blindly via email (the interviewee answered ignoring the methodology, WS or AHP). Thus, the researchers determined which method best expressed the preferences of each stakeholder according to their criteria.

### Working group 4

WG4 again weighted the criteria and sub-criteria, but only by the method (WS) that best expressed the preferences of WG3 in the survey. This new weighting was performed to check the consistency of WG3’s results. WG4 reproduced the multicriteria appraisal process of WG3 via the Internet. It was composed of a minimum of 15 patients, 15 clinicians, and 15 managers. Thus, the method sequentially included a first baseline weighting (WS), structured deliberation, and a second weighting exclusively using the methodology preferred by WG3 (WS or AHP). An ad hoc website was designed in HTML5 with CSS, JavaScript and AJAX on the client side, and PHP with MySQL on the server side, tools that met the necessary requirements imposed by the methodology. The discussion via Internet was anonymous, but the category of the stakeholder was shown (patient, clinician, or manager).

### Working group 5

Finally, WG5 integrated two independent academic experts in MCDA, who analysed the results. The statistical study was carried out using SPSS software and consisted of the following phases: (i) analysis of face-to-face results by stakeholder category and by methodology (WS vs. AHP); and (ii) analysis of significant differences between face-to-face and Internet results by stakeholder category, a T-test of means and ANOVA methods have been used for statistical analysis and significance. A Bonferroni test have been used to prevent data from incorrectly appearing to be statistically significant, if necessary.

## Results

The bibliographic search carried out by WG1 resulted in 17 articles that met the requirements imposed by the MCDA methodology. WG1 identified five criteria: evidence-based variables (EBVs), morbidity, mortality, patient reported outcome measures (PROMs), and patient reported experience measures (PREMs). PROMs are health outcomes that capture symptoms, functional status, and quality of life. PREMs measure aspects related to the humanity of care, such as the dignity of care or communication with healthcare personnel [20]. In turn, the EBV criteria included four sub-criteria: dialysis dose, haemoglobin concentration, mineral and bone disease, and type of vascular access.

After the qualitative analysis, WG2 confirmed all of the established criteria and sub-criteria and asked WG1 to include a new sub-criterion within the EBVs, the “ratio of bacteraemia related to the catheter”. This modification was detected independently by the two analysts in the meeting as a need perceived by the three stakeholder groups. This indicator was considered by all of the groups to be an essential element in the safety of patients. WG1 included it when fulfilling all of the established requirements. Table 1 reflects the final structure of the approved criteria and sub-criteria and their definitions. The criteria are defined positively to allow their aggregation and the construction of a performance matrix.

**Table 1:** Criteria and sub-criteria established for haemodialysis treatment

Criteria	Sub-criteria	Definition
Evidence-based variables		Recommendation level 1 in GRADE clinical guidelines
	Type of vascular access	% of patients with functioning autologous vascular access
	Dialysis dose	% of patients with Kt/v > 1.4 (adequate dose)
	Haemoglobin concentration	% of patients with haemoglobin of 11–13 g/dl
	Ratio of bacteraemia related to the catheter	% of patients without bacteraemia in the unit in a period of one year
	Mineral and bone disease	% of patients with calcium 8.4–10 mg/dl and phosphorus 2.5–4.5 mg/dl
Morbidity, annual		% of patients without hospitalization in a period of one year
Mortality, annual		% survival in a period of one year
PROMs		% of the SF-36 quality of life survey (MCS and PCS)
PREMs		% of DCQ satisfaction survey

*PROM* patient reported outcome measure, *PREM* patient reported experience measure, *GRADE* grading of recommendations assessment, development, and evaluation, *Kt/v*: dialysis adequacy was calculated with the single pool Daugirdas II method, *MCS* Mental component summary from SF-36, *PCS* Physical component summary from SF-36, *DCQ* quality of care in Dialysis Centre Questionnaire

Table 2 shows the relative importance of the criteria (from 0 to 100) and sub-criteria (from 0 to 100) expressed by the members of WG3 in the second weighting. The aggregate results of WG3 (A, B, and C) are shown by both methodology (WS and AHP) and category of stakeholder (patients, clinicians, and managers). The first weighting and the results disaggregated by WG3 (A, B, and C) are not collected to simplify the table. For patients, the criterion most valued by both methodologies was PROMs (WS 28.33 and AHP 36.26) and it was superior to that of the other stakeholders. For clinicians and managers, the most valued criteria were PROMs and EBVs, depending on the methodology used. Among the sub-criteria, the type of vascular access was the most valued criterion with both methodologies by all stakeholders. The analysis of face-to-face results by stakeholder category and methodology (WS vs. AHP) using ANOVA showed only minor significant differences between stakeholders for the criteria of morbidity and mineral and bone disease. Importantly, ANOVA showed that the groups were not comparable due to the small sample.

**Table 2:** Aggregated results of Working Group 3 (A, B, and C)

			Patients	Clinicians	Managers	Total
			(*n* = 9)	(n = 9)	(*n* = 8)	(*n* = 26)
Criteria	Sub-criteria	Methodology				
EBV		WS	26.11 (10.83)	26.66 (7.50)	24.37 (10.16)	25.77 (9.24)
		AHP	20.31 (15.06)	24.62 (17.60)	29.50 (22.25)	24.63 (18.02)
	Type of vascular access	WS	25.55 (8.46)	26.67 (4.33)	28.75 (5.18)	26.92 (6.17)
		AHP	41.66 (11.38)	40.42 (11.41)	31.98 (14.37)	38.25 (12.63)
	Dialysis dose	WS	26.11 (8.21)	20.56 (3.01)	21.87 (5.94)	22.88 (6.35)
		AHP	18.63 (10.45)	21.17 (11.19)	19.75 (11.95)	19.85 (10.78)
	Haemoglobin concentration	WS	16.11 (4.17)	18.33 (2.50)	15.62 (4.17)	16.73 (3.73)
		AHP	13.36 (6.18)	11.03 (7.57)	10.61 (7.00)	11.71 (6.78)
	Ratio of bacteraemia related to the catheter	WS	16.11 (8.21)	21.67 (7.50)	25.00 (3.78)	20.77 (7.57)
		AHP	19.93 (11.95)	23.61 (10.91)	19.29 (20.68)	21.01 (14.40)
	Mineral and bone disease	WS	16.11 (6.97)	12.78 (7.12)	8.75 (3.54)	12.69 (6.67)
		AHP	6.42 (3.47)	3.77 (0.99)	18.36 (14.22)	9.18 (10.05)
Morbidity, annual		WS	15.56 (7.68)	18.89 (5.46)	18.37 (8.43)	17.57 (7.12)
		AHP	23.06 (7.98)	13.15 (9.35)	15.72 (9.88)	17.38 (9.72)
Mortality, annual		WS	12.22 (7.55)	18.89 (6.50)	20.00 (9.26)	16.92 (8.26)
		AHP	13.76 (13.75)	20.51 (20.71)	20.68 (20.02)	18.23 (17.91)
PROMs		WS	28.33 (5.00)	21.67 (4.33)	26.25 (7.44)	25.38 (6.15)
		AHP	36.26 (16.76)	31.93 (12.45)	23.33 (13.23)	30.78 (14.75)
PREMs		WS	17.78 (6.18)	13.89 (3.33)	11.00 (2.56)	14.34 (5.05)
		AHP	6.61 (3.35)	9.77 (5.78)	10.75 (11.94)	8.98 (7.58)

The disaggregated results are shown by category of stakeholder (patient, clinician, and manager) and by methodology (WS and AHP). The first weight is not given to simplify the table. Data are given as mean (SD)

*EBV* evidence-based variables, *WS* weighted sum, *AHP* analytic hierarchy process, *PROM* patient reported outcome measure, *PREM* patient reported experience measure, *SD* standard deviation

The results of the individual survey of WG3 are shown in Table 3. The majority (61.5%) expressed a preference for the WS method, and we decided to continue the investigation in WG4 with the WS method only.

**Table 3:** Result of the survey of the members of Working Group 3 (A, B, C) in which they were asked about the method that best reflects their preferences (WS vs. AHP)

Preference	Criteria	Criteria	Sub-criteria	Sub-criteria
	Stakeholder (n)	Stakeholder (%)	Stakeholder (n)	Stakeholder (%)
WS	16	61.5	16	61.5
AHP	6	23.1	7	26.9
None	0	0.0	0	0.0
Both	1	3.8	0	0.0
No answer	3	11.5	3	11.5
Total	26	100.0	26	100.0

*WS* weighted sum, *AHP* analytic hierarchy process

Table 4 shows the weights of the criteria and sub-criteria given by the members of WG4 disaggregated by category of stakeholder (patients, clinicians, and managers). In the Internet group (WG4), ANOVA only detected small significant differences between stakeholders for PROMs (*P* = 0.047). The Bonferroni test showed that the differences detected were only between patient and managers (*P* = 0.043). These data were not included in the table for simplicity.

**Table 4:** Weighting of the criteria and sub-criteria made by Working Group 3 (A, B, C) face-to-face and Working Group 4 via the Internet using the WS methodology and its comparison

		WG3	WG4		WG3	WG4		WG3	WG4		WG3	WG4	
		Patients	Patients		Clinical	Clinical		Managers	Managers		Total	Total	
		(*n* = 9)	(*n* = 16)		(*n* = 9)	(*n* = 24)		(*n* = 8)	(*n* = 19)		(*n* = 26)	(*n* = 59)	
Criteria	Sub-criteria	Mean (SD)	Mean (SD)	*P*-value	Mean (SD)	Mean (SD)	*P*-value	Mean (SD)	Mean (SD)	*P*-value	Mean (SD)	Mean (SD)	*P*-value
Evidence-based variables		26.11 (10.83)	22.81 (6.32)	0.343	26.66 (7.50)	24.25 (10.26)	0.525	24.37 (10.16)	25.42 (10.67)	0.816	25.77 (9.24)	24.24 (9.40)	0.489
	Type of vascular access	25.55 (8.46)	30.94 (8.98)	0.156	26.67 (4.33)	28.04 (8.21)	0.638	28.75 (5.18)	28.68 (9.10)	0.985	26.92 (6.17)	29.03 (8.65)	0.265
	Dialysis dose	26.11 (8.21)	22.06 (7.96)	0.24	20.56 (3.01)	25.8 (6.20)	0.045*	21.87 (5.94)	22.11 (6.52)	0.932	22.88 (6.35)	23.31 (6.86)	0.786
	Haemoglobin concentration	16.11 (4.17)	15.37 (5.31)	0.724	18.33 (2.50)	15.46 (3.93)	0.051	15.62 (4.17)	15.79 (6.06)	0.909	16.73 (3.73)	15.54 (4.04)	0.204
	Ratio of bacteraemia related to the catheter	16.11 (8.21)	17.50 (9.13)	0.709	21.67 (7.50)	20.21 (4.81)	0.512	25.00 (3.78)	21.84 (6.06)	0.186	20.77 (7.57)	20.00 (7.71)	0.671
	Mineral and bone disease	16.11 (6.97)	14.12 (6.75)	0.492	12.78 (7.12)	11.21 (4.91)	0.476	8.75 (3.54)	11.57 (5.28)	0.179	12.69 (6.67)	12.12 (5.62)	0.685
Morbidity, annual		15.56 (7.68)	14.69 (8.65)	0.805	18.89 (5.46)	16.87 (5.67)	0.366	18.37 (8.43)	20.26 (6.20)	0.522	17.57 (7.12)	17.37 (6.99)	0.904
Mortality, annual		12.22 (7.55)	14. 6 (6.88)	0.541	18.89 (6.50)	14.42 (5.59)	0.059	20.00 (9.26)	16.42 (8.20)	0.328	16.92 (8.26)	14.96 (6.82)	0.256
PROMs		28.33 (5.00)	30.00 (8.94)	0.613	21.67 (4.33)	25.62 (6.96)	0.123	26.25 (7.44)	23.26 (8.00)	0.375	25.38 (6.15)	26.05 (8.16)	0.709
PREMs		17.78 (6.18)	18.44 (10.28)	0.863	13.89 (3.33)	18.33 (5.93)	0.025*	11.00 (2.56)	14.10 (5.34)	0.132	14.34 (5.05)	17.20 (7.40)	0.076

The results are disaggregated and compared by category of interested party (patient, clinician, manager). Data are given as media (DE)

*EBV* evidence-based variable, *WG* working group, *WS* weighted sum, *AHP* analytic hierarchy process, *PROM* patient reported outcome measure, *PREM* patient reported experience measure, *SD* standard deviation

Ratio of bacteraemia RC, Ratio of bacteraemia related catheter

*p < 0.05

Table 4 also presents the results of WG3 disaggregated in the same way. A comparison is made between both WGs (WG3-A, B, C vs. WG4). The table shows that there are no significant differences between the two groups (face-to-face vs. Internet) for most of the results. The only differences detected are in two parameters in the category of clinicians: EBVs (dialysis dose, *P* = 0.045) and PREMs (*P* = 0.025).

By presenting only minor differences between WG3 and WG4 (face-to-face vs. Internet), both groups were considered comparable and their results added. After the addition of WG3 and WG4, ANOVA was performed to detect differences between stakeholders. The results showed significant differences that were confirmed by the Bonferroni test for the following criteria and groups: morbidity, patients vs. managers (P = 0.045); PROMs, patients vs. clinicians (*P* = 0.042) and patients vs. managers (*P* = 0.034); and PREMs, patients vs. managers (*P* = 0.023) and clinicians vs. managers (P = 0.042). For the sub-criteria and groups, the differences were ratio of bacteraemia, patients vs. managers (*P* = 0.007), and mineral and bone disease, patients vs. managers (*P* = 0.036; Table 5).

**Table 5:** Aggregate results of the Working Group 3 and Working Group 4 and comparison of differences between the stakeholder groups

		Patients	Clinicians	Managers	Patients/ Clinicians	Patients/ Managers	Clinicians/ Managers
		(*n* = 25)	(*n* = 33)	(*n* = 27)			
		Mean (SD)	Mean (SD)	Mean (SD)	*P*-value	*P*-value	*P*-value
Criteria	Sub-criteria						
Evidence-based variables		24.00 (8.16)	24.91 (9.53)	25.11 (10.34)	1.000	1.000	1.000
	Type of vascular access	29 .00 (9.01)	27.66 (7.32)	28.70 (8.03)	1.000	1.000	1.000
	Dialysis dose	23.52 (8.12)	23.84 (5.83)	22.03 (6.24)	1.000	1.000	0.903
	Haemoglobin concentration	15.64 (4.85)	16.24 (3.79)	15.74 (3.31)	1.000	1.000	1.000
	Ratio of bacteraemia related to the catheter	17.00 (8.66)	20.60 (5.57)	22.77 (5.60)	0.131	0.007*	0.633
	Mineral and bone disease	14.84 (6.75)	11.63 (5.52)	10.74 (4.94)	0.116	0.036*	1.000
Morbidity, annual		15.00 (8.16)	17.42 (5.60)	19.70 (6.82)	0.551	0.045*	0.604
Mortality, annual		13.40 (7.03)	15.63 (6.09)	17.48 (8.51)	0.735	0.133	0.98
PROMs		29.4 (7.68)	24.54 (6.54)	24.15 (7.81)	0.042*	0.034*	1.000
PREMs		18.20 (8.89)	17.48 (5.75)	13.18 (4.86)	1.000	0.023*	0.042*

*PROM* patient reported outcome measure, *PREM* patient reported experience measure, *SD* standard deviation

*p < 0.05

Finally, Table 6 includes a weighting proposal for each criterion aimed at a hypothetical evaluation of the results of haemodialysis centres. It also presents the mean standard deviation of each criterion as a reference value to conduct a sensitivity study of said evaluation. This table has been prepared with the results from WG4. Thus, the EBVs would have a weight of 24.24 points out of the total 100 in the evaluation.

**Table 6:** Proposal of weights for each criterion in a hypothetical evaluation of dialysis centres and their standard deviation

Criteria	Sub-criteria	Weight	SD
Evidence-based variables		24.24	9.40
	Type of vascular access	29.03	8.65
	Dialysis dose	23.31	6.86
	Haemoglobin concentration	15.54	4.04
	Ratio of bacteraemia related to the catheter	20.00	7.71
	Mineral and bone disease	12.12	5.62
Morbidity, annual		17.37	6.99
Mortality, annual		14.96	6.82
PROMs		26.05	8.16
PREMs		17.20	7.40

*PROM* patient reported outcome measure, *PREM* patient reported experience measure, *SD* standard deviation. The addition of weights is 99.83, instead of 100, due to rounding error

## Discussion

Our study shows that there are different perceptions and valuations among the different criteria for evaluating haemodialysis. Thus, patients give greater importance to PROMs than clinicians and managers, and this happens with all three estimation methods used (face-to-face: WS and AHP, and via Internet). The results corroborate a finding that has already been revealed in previous research using other methodologies [21, 22]. Mortality also has a differentiated weighting: lower for patients and higher for clinicians and managers. Despite these differences in assessment among the stakeholders, only recently has the need to include the patient’s perspective in a routine and explicit way been emphasized [23–26]. PROMs are a priority for patients and other stakeholders, reflecting their preferences, and should be systematically considered in evaluation systems.

The objective of the evaluation of health services is threefold: (i) to quantify the quality of the service provided; (ii) allow specific programs and activities aimed at improvement to be established; and (iii) enable accountability and citizen control of the services provided. Due to the transcendence of these objectives, it is an indispensable duty to have a comprehensive evaluative methodology that is valid, participatory, acceptable, and feasible.

The multicriteria methodology is a formal deliberative discussion procedure that uses explicit criteria. The method incorporates the perspective of the stakeholders in determining the preferences of the process studied. The preferences and intangible aspects are synthesized in the criteria and their weights. The mathematical expression of the preferences constitutes the performance matrix, with which an aggregation function of the results can be constructed, capable of adding these in a single expression value. The use of a performance matrix of indicators, such as the one proposed in Table 6, provides a measure of proportionality and uncertainty for each criterion that reflects the values of the stakeholders. The matrix can be useful to provide validity, legitimacy, and transparency to an analysis of the results and to the elaboration of clinical guidelines based on the values and preferences of the stakeholders [27].

Health services are made up of a multitude of components that interact with one another in a frequently unpredictable way. They constitute “complex adaptive systems” influenced by biochemical, cellular, physiological, genetic, pathological, pharmacological, organizational, psychological, social, cultural, economic, and political aspects that determine considerable uncertainty in the face of individual and collective decisions [13]. In addition, multiple cognitive limitations in information processing interfere with clinical and organizational decision making [28]. It has been postulated that the conceptualization of the health environment as a complex system can help in its understanding and improvement, by banishing simplistic paradigms of linear thinking [29]. In this context, the use of an evaluation model endowed with a multiple, transdisciplinary, and reflective perspective can constitute a tool to help assess the results and make decisions closer to the complexity of the real world.

The methodology allows a rational hierarchy of complex elements, such as the different EBVs. In multicriteria deliberations, all EBVs were subordinated to the type of vascular access, which is the most valued sub-criterion, and this subordination was widely accepted by the various stakeholders. The reason for this is that adequate vascular access improves the results of the other four EBVs, but this property does not happen the other way around for any of the four variables. The capture of nuances of a multilateral relationship between indicators helps characterize them, and their knowledge facilitates a judicious exercise of clinical practice.

The implications of this study on the evaluation of centres are important. Health processes are complex systems in which no individual actor is knowledgeable about the whole of their operation. Therefore, it is essential to design evaluation procedures that consider multiple perspectives and are based on broader societal involvement to effectively improve our knowledge of the process. With this study, we have determined the relevant outcomes of haemodialysis, quantifying their relative importance and potential degree of uncertainty. An evaluation of dialysis centres using this methodology may be more accurate and legitimate. In addition, this evaluation methodology can be reproduced and used in other homogeneous clinical processes, such as kidney transplant, hip replacement, or many others. To enable meaningful comparisons across centres, the case-mix variables of those centres need to be appropriately adjusted.

This evaluation, which considers the complexity of health organizations, may be an effective tool in helping clinical and management decision-making.

The study has several limitations. First, although there is an epistemological basis for the knowledge generated, the performance matrix could create a different structure in another cultural environment. As has been suggested, the subject is rooted in a social order that is a source of subjectivism [13]. It would be important to validate the weighting of the criteria in a different cultural environment before their practical application in it. Second, although the concept of PREMs is defined, there is no consensus about the use of questionnaires in practice in haemodialysis [25]. For this reason, the reflection of the group is adequate from a conceptual perspective, but imprecise when going down into the detail of the content of the questionnaires due to their heterogeneity. Despite the limitations of the study, we think that an evaluative approach that considers these indicator weights is more consistent than a perspective that does not discriminate between indicators, as it better reflects the values ​​of the stakeholders.

## Conclusions

Our results suggest that the different types of stakeholders manifest distinct preferences among indicators, and this happens consistently when captured by different methodologies. Thus, patients have a greater preference for indicators related to PROMs than clinicians and managers, and this consideration must be incorporated into the assessment of health services. The use of a multicriteria methodology endowed with a multifocal, transdisciplinary, and reflective perspective allows us to determine the relative importance and uncertainty of the various evaluation indicators, as a reflection of the values of the stakeholders and society. The inclusion of values in the evaluation, through a performance matrix, could help with clinical and organizational decision-making in a complex system.

## Data Availability

All data supporting the results of the study are available from the corresponding author on reasonable request.

## Abbreviations

DOPPS: *Dialysis Outcomes and Practice Patterns Study*
MCDA: Multicriteria decision analysis
WGs: Working groups
WS: Weighted sum
AHP: Analytic hierarchy process
EBVs: Evidence-based variables
PROMs: Patient reported outcome measures
PREMs: Patient reported experience measures
